# Non Hodgkin lymphoma in Lebanon: a retrospective epidemiological study between 1984 and 2019

**DOI:** 10.1186/s12889-021-11840-3

**Published:** 2021-10-09

**Authors:** Elsie Touma, Leony Antoun, Souheil Hallit, Fadi Nasr, Marcel Massoud, Radwan El Othman, Georges Chahine

**Affiliations:** 1 Faculty of Medicine and Medical Sciences, Holy Spirit University of Kaslik (USEK), Jounieh, Lebanon; 2 Department of Hematology-Oncology, University Hospital Center-Notre Dame Des Secours, Jbeil, Lebanon; 3 Research Department, Psychiatric Hospital of the Cross, Jal Eddib, Lebanon; 4 Faculty of Medicine, Saint-Joseph University, Beirut, Lebanon; 5 Department of Hematology-Oncology, University Hospital Center- Hotel-Dieu de France, Beirut, Lebanon; 6 Department of Hematology-Oncology, Mont-Liban Hospital, Hazmieh, Lebanon; 7 Department of Hematology-Oncology, Bellevue Medical Center, Mansourieh, Lebanon

**Keywords:** Non Hodgkin lymphoma, Epidemiology, Subtypes, Lebanon

## Abstract

**Background:**

Lymphomas are ranked as the fifth most common cancer in Lebanon. There is concern about the need of information regarding the prevalence of lymphoid neoplasm particularly Non-Hodgkin lymphoma (NHL) subtypes in the Lebanese population. This study intended to establish a descriptive status of NHL histological subtypes distribution in Lebanon thus identifying the most common types, knowing that the literature is poor regarding the distribution of lymphoid malignancies particularly NHLs in Lebanon.

**Methods:**

A bicenter retrospective descriptive study was performed. Patients aged above 18, diagnosed with NHL between January 1984 and March 2019 and registered in two Lebanese Medical centers were included in this study; 699 medical files were reviewed and the baseline characteristics of the disease were collected. Histological classification was based on the Working Formulation (WF) and World Health Organization (WHO) classification systems, whereas staging was based on the Ann Arbor system. Disease status was monitored with imaging studies.

**Results:**

The mean age at diagnosis was 53.52 ± 17.46 years in the studied population, with 380 (54.4%) males and 319 (45.6%) females. B-cell lymphoma (BCL) accounted for 86.3% while T-cell neoplasms accounted for 13.7%. The most common subtype was diffuse large B-cell lymphoma (DLBCL) (54%) followed by follicular lymphoma (FL) (17.2%). Mantle cell lymphoma (MCL) represented 3% of all BCL and small lymphocytic lymphoma (SLL) comprised less than 2%. Mucosa-associated lymphoid tissue (MALT) and Burkitt’s lymphomas represented 3 and 1.7% respectively. 36.5% of the patients had extranodal disease at diagnosis. High-grade tumor represented 80.1% with 33.1% stage IV disease.

**Conclusion:**

These observations indicate that the epidemiological patterns of NHLs in Lebanon were comparable to Western countries. Aggressive lymphomas account for the majority of NHLs in Lebanon.

## Background

Lymphomas are ranked as the fifth most common cancer in Lebanon as suggested by several epidemiological studies done at the time [1, 2]. Non Hodgkin Lymphoma (NHL) patients constitute a group of interest to many epidemiologists [1]. In contrast to the adequate information available on the epidemiology of NHL from developed nations, such data from developing countries is scattered [3]. To date, there is concern about the need of information regarding the prevalence of lymphoid neoplasm subtypes in the Lebanese population [1]. Worldwide, the incidence of NHL is rising, mostly in older population. This is also the case in Lebanon [2, 4].

NHLs consist of a diverse group of hematologic malignancies deriving from mature or immature lymphocytes (B, T or NK). B-cell lymphomas (BCLs) account for 80 to 85% of the cases especially in the Western world and United States (US), and T-cell lymphomas (TCLs) accounts for the rest (15 to 20%) [5]. In Lebanon, most NHL cases are of B-cell origin [1]; however, histologic subtypes can vary in different parts of the world [6]. The subclassification of the disease underwent significant changes due to improvements in the molecular biology and cellular genetics field especially with the introduction of anti-CD 20 antibody that has supplemented the diagnosis and treatment options available for BCL [7]. Obviously, advances in treatment modalities contributed to improvement in survival for several NHL subtypes. Besides patients characteristics, socioeconomic factors influence survival because it is documented that in countries with higher income, the 5 year overall survival (OS) is almost 80% and much lower in middle or low income setting, with respect of the difference in age and histology [8].

NHL is the most prevalent hematopoietic neoplasm ranking seventh in frequency among all cancers [9]. Diffuse large B cell lymphoma (DLBCL) type constitutes 40% of lymphomas, and is more diagnosed among middle-aged men, while follicular lymphoma (FL) accounts for about 20%. These subtypes are most frequent in North America and Europe [8]. The NHL subtypes variation in each country appears to be related to population characteristics and environmental factors. For example, the incidence in the US is greater than other countries with a predominant nodal disease. Although NHL incidence is relatively low in Asian countries, Asians generally present with a higher proportion of TCLs [8]. In Africa, there is insufficient data available but the most documented type is Burkitt’s lymphoma [10]. As for the Arab countries, NHL is common in Egypt, Kuwait, Oman, and Saudi Arabia, accounting for about 10% of all cancers [10]. It is the fourth major cause of cancer incidence in Egypt, Oman, Qatar and Bahrain [11].

The purpose of this study was to evaluate and recognize the most prevalent subtypes of NHL in Lebanon. Because there is scarce multi centric data demonstrating the epidemiological patterns of NHL occurrence in Lebanon and knowing that there is unpublished data and only a small percentage of all newly Lebanese cancer cases are diagnosed in tertiary hospitals, there is constant need of studies and timely documentations of the disease prevalence. This may serve as an added value to the current literature and a basis for future studies. Hence, we decided to conduct such study at our centers and evaluate the clinical features of NHL hypothesized to parallel those seen in Western countries.

## Methods

### Study design

A bicenter, retrospective descriptive study was performed in Lebanon. Patients aged above 18, diagnosed with NHL between January 1984 and March 2019 inclusive and registered in two Lebanese Medical centers (University Hospital Center-Notre Dame Des Secours, University Hospital Center-Hotel Dieu de France) were included in this study. The medical files of 699 patients were extracted from the hospital archive. The following characteristic variables were collected from the records: age at diagnosis, sex, histological subtype, B or T type, extent and sites of the disease (nodal/extra nodal), stage at diagnosis, first line chemotherapy administrated, use of radiotherapy (RT). Pathologic diagnosis was documented on the basis of histologic confirmation by tissue biopsy, immunohistochemical studies using a panel of antibodies depending on the morphology of the biopsy and flow cytometry. The pathological documentation is similar between all subgroups and additional evaluations using special techniques were used to elucidate specific types (to identify specific chromosomal translocations and molecular phenotypes). Patients diagnosed before 2001 were classified histologically according to the Working Formulation classification [12] and those diagnosed after 2001 were classified according to the WHO classification [13].

### Instrument for data collection

For the purpose of data collection from the medical records, we used a consistent series of questions in the form of an excel table sheet to determine the variables and assess the baseline characteristics associated with each NHL case. These information included as follows: patient’s name, sex, year of diagnosis, age at diagnosis, histologic type, B or T type, nodal or extranodal, grade, stage at diagnosis, first line chemotherapy given, response to treatment, use of RT and last follow up date.

### Sample size

According to the Epi-info software, based on a 6 million inhabitants in Lebanon, a 4% worldwide prevalence of NHL^10^, a 5% margin of error (i.e. 95% confidence interval) and a design effect of 5, the minimal sample size needed was 295.

### Staging

Clinical stage was defined according to the Ann Arbor classification [14]. In stage 1, only one node or a group of contiguous nodes are involved, while in stage 2 two or more group of nodes being on the same side of the diaphragm are involved. Stage 3 and 4 are both advanced stages where nodes on both sides of the diaphragm or additional non-contiguous extralymphatic involvement are seen respectively [15]. The nodal localization considers the involvement of lymph nodes, spleen, thymus and Waldeyer’s ring while the extranodal one considers other organs involvement [7].

### Statistical analysis

Data entry and analysis were performed using the Statistical Package for the Social Sciences (SPSS) version 23. The validity and reliability of the data were checked by an independent person (not related to the research team) by randomly selecting some patients’ files and verifying that the data entered did not include any mistakes. The mean OS was calculated from diagnosis to death from any cause. The Chi-square test was used to compare categorical variables, whereas the Student t test was used to compare two means. *P* < 0.05 was considered statistically significant.

## Results

### Characteristics of the sample

The mean age at diagnosis was 53.52 ± 17.46 years in the studied population, with 380 (54.4%) males and 319 (45.6%) females. The mean age for males was 53.56 ± 17.95 years, and for female 53.48 ± 16.87 years (*p* = 0.954).

**Fig. 1:**
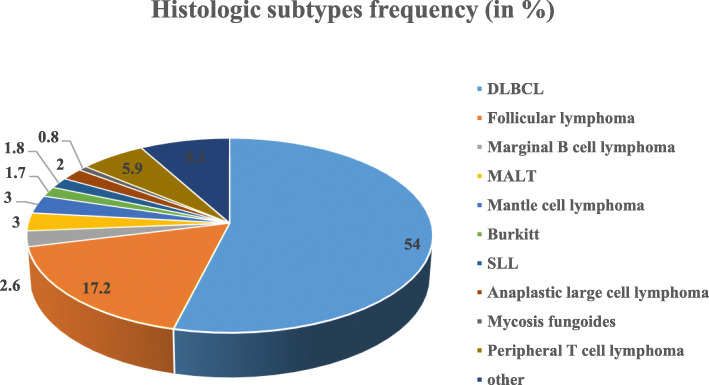
Frequency in percentages of the common histologic subtypes

BCLs accounted for 86.3% while T-cell neoplasms accounted for 13.7%. The most common subtype was DLBCL (54%) followed by FL (17.2%). Mantle cell lymphoma (MCL) represented 3% of all BCL and small lymphocytic lymphoma (SLL) comprised less than 2%. Mucosa-associated lymphoid tissue (MALT) and Burkitt’s lymphomas represented 3 and 1.7% respectively. (Fig. 1).

In addition, 36.5% of the patients had extranodal disease at diagnosis. High-grade tumor represented 80.1% with 33.1% stage IV disease.

RT was performed in 18.6% of the patients. The death rate was 25.5%, with a mean overall survival of 121.89 ± 0.89 months. It is of note that the 5-year survival rate was 63.2% (Table 1).

**Table 1:** Sociodemographic and other characteristics of the patients diagnosed with non-Hodgkin lymphoma (*N* = 699)

Variable	N (%)
**Gender**	
Female	319 (45.6%)
Male	380 (54.4%)
**Cell type NHL**	
B	603 (86.3%)
T	96 (13.7%)
**Lesion at diagnosis**	
Nodal	442 (63.2%)
Extranodal	254 (36.3%)
Missing data	3 (0.5%)
**Staging**	
1	97 (13.9%)
2	179 (25.6%)
3	167 (23.9%)
4	219 (31.3%)
Missing data	37 (5.3%)
**Radiotherapy**	
No	563 (80.5%)
Yes	129 (18.5%)
Missing data	7 (1.0%)
**Death**	
No	521 (74.5%)
Yes	178 (25.5%)
**Age (in years)**	53.52 ± 17.46

### Comparison between genders

A significantly higher percentage of males received the dose dense regimen compared to RCHOP or other regimens. No significant difference was found between genders in terms of death rate, NHL cell type, lesion at diagnosis, NHL staging, radiotherapy and age (Table 2).

**Table 2:** Comparison of the studied variables according to gender

Variable	Male	Female	***p***	Statistical test used
**Death**			0.108	Chi-square
No	274 (52.6%)	247 (47.4%)		
Yes	106 (59.6%)	72 (40.4%)		
**Cell type NHL**			0.069	Chi-square
B	320 (53.1%)	283 (46.9%)		
T	41 (65.1%)	22 (34.9%)		
**Lesion at diagnosis**			0.188	Chi-square
Nodal	233 (52.7%)	209 (47.3%)		
Extranodal	147 (57.9%)	107 (42.1%)		
**Staging**			0.427	Chi-square
1	55 (56.7%)	42 (43.3%)		
2	95 (53.1%)	84 (46.9%)		
3	98 (58.7%)	69 (41.3%)		
4	111 (50.7%)	108 (49.3%)		
**First line chemotherapy**			**0.036**	Chi-square
RCHOP	146 (51.4%)	138 (48.6%)		
Dose dense	60 (66.7%)	30 (33.3%)		
Other regimens	96 (57.1%)	72 (42.9%)		
**Radiotherapy**			0.763	Chi-square
No	306 (54.4%)	257 (45.6%)		
Yes	72 (55.8%)	57 (44.2%)		
**Age (in years)**	53.56 ± 17.95	53.48 ± 16.87	0.954	Student t test

Numbers in bold indicate significant *p*-values.

### Comparison by age

When comparing the variables by age (≤64 vs ≥65 years), the results showed that a significantly lower percentage of patients who received RT was aged 65 years or more. No significant association was found with all other variables (Table 3).

**Table 3:** Comparison of the studied variables according to age (≤64 and ≥ 65 years)

Variable	≤ 64 years	≥ 65 years	***p***	Statistical test used
**Gender**			0.737	Chi-square
Male	267 (55.1%)	110 (53.7%)		
Female	218 (44.9%)	95 (46.3%)		
**Death**			0.847	Chi-square
No	363 (74.8%)	152 (74.1%)		
Yes	122 (25.2%)	53 (25.9%)		
**Cell type NHL**			0.09	Chi-square
B	411 (89.2%)	184 (93.4%)		
T	50 (10.8%)	13 (6.6%)		
**Lesion at diagnosis**			0.571	Chi-square
Nodal	303 (62.6%)	133 (64.9%)		
Extranodal	181 (37.4%)	72 (35.1%)		
**Staging**			0.729	Chi-square
1	69 (15.0%)	28 (14.3%)		
2	126 (27.4%)	52 (26.5%)		
3	110 (23.9%)	55 (28.1%)		
4	155 (33.7%)	61 (31.1%)		
**First line chemotherapy**			0.101	Chi-square
RCHOP	192 (50.5%)	89 (56.7%)		
Dose dense	72 (18.9%)	18 (11.5%)		
Other regimens	116 (30.5%)	50 (31.8%)		
**Radiotherapy**			**0.01**	Chi-square
No	380 (78.8%)	178 (87.3%)		
Yes	102 (21.2%)	26 (12.7%)		

Numbers in bold indicate significant *p-*values

## Discussion

NHL encompasses various lymphoid neoplasms with different clinical and biological profiles. As evidenced in our study, NHL is commonly observed among middle-aged males with B-cell type representing more than two third of the cases. DLBCL and FL were the predominant subtypes. Patients in this study mainly presented at an advanced stage thus suggesting the probable effect of poor access to medical care.

NHL disease among Lebanese patients appear to be more prevalent in adult males, a tendency also seen in the western countries [12]. According to the literature, males are more affected of NHL than females with approximately 30% higher incidence [15]. In fact, several researchers investigated how sex hormones modulate lymphoid neoplasms. The reduced rate of NHL among females is best explained by the effect of estrogen on modulating tumoral cell proliferation [16]. In their study, Yakimchuk et al. investigated the anti-proliferative effect of estrogen through estrogen receptor β (ERβ) signaling [17]. Furthermore, in a study done in 2016 by Perry and colleagues evaluating the frequencies of NHL subtypes in five developing regions of the world, there was a significant difference in the sex distribution with a notably higher number of males in contrast to the developed world [18]. This could suggest the presence of sex inequality when seeking medical care in these countries and consequently women being underdiagnosed with lymphomas [19]. Our results were in accordance with previous studies stating the predominance of NHL in males. Further studies stratifying patients according to their socioeconomic status are warranted to assess whether this factor impact access to healthcare in our country.

In this study, the mean age of the patients is 53.52 years, which is moderately higher than that of patients from Arab countries: Saudi Arabia (46 years) (Koriech and Al-Kuhaymi, 1994) [20], Jordan (44 years) from 1996 till 1999 (Almasri et al., 2003) [21] and Egypt (51.6 years) from 1995 to 2004 (Abdel-Fattah et al., 2007) [22]. In northern India the mean age was 47 years (Sandhu et al., 2018) [4] from 1997 to 2000. In the US, between 2012 and 2016, the mean age at diagnosis of NHL was 67 years [8]. In South East Asia, from 2007 to 2014 the mean age was 56 years (Intragumtornchai et al., 2018) [23].

B-cell type represented 86.3% of NHL cases in Lebanon, which is in accordance with the worldwide reported rates (80–90%), except for the Eastern countries where T-cell type rate is higher [7]. The proportion of TCL is 13.7% of all cases in our study. This is comparable to the results in western countries where TCL proportion does not exceed 10% in England [24], 12% in France (Troussard et al., 2009) [25] and 15% in the US [26]. However, this percentage of TCL is very low when compared to China (30% of all NHL) (Yang et al., 2011) [27] and Japan (27%) (Aoki et al., 2008) [28]. Regarding the higher frequency of TCLs in Asia than the rest of the world, this appears to be related to the HTLV-1 virus infection which is more prevalent in Japan and the Caribbean countries [7]. In addition when stratifying according to gender, the observed proportion of TCLs among male cases was 65.1% (vs. 34.9% among females). This is in accordance with results from the Surveillance, Epidemiology, and End Results (SEER) where the reported incidence of TCLs showed a higher male/female ratio from 1992 to 2001 in contrast to other subtypes [18].

In Lebanon, a one-year national study of 227 cases of lymphomas classified according to the 2001 WHO classification of malignant lymphomas has been published by Otrock et al. in 2013. The results were notable for 88% of BCLs and 9% of TCLs. These proportions are in part similar to the observed results in our study [29].

High-grade tumor predominated in 80.1% of cases, with DLBCL and FL being the most common subtypes. DLBCL comprises 54% of all cases. Similar frequency was noted in Jordan (53%) [23] and Algeria (52.8%) (Boudjerra et al., 2015) [30]. FL represents 17.2% of the cases, in comparison to 15.9% in the UK (Smith et al., 2015) [31] and 17% in the US (Chihara et al., 2014) [32] while a rate of 7% was noted in Saudi Arabia (Akhtar et al., 2009) [33]. SLL comprised less than 2% of NHL cases in contrast to 15% in the USA [16]. MCL represents 3% of all BCL, which is close to the rate seen in Saudi Arabia (2%) (Akhtar et al., 2009) , USA (3%) (Wu et al., 2009) [34] and France (4%) (Troussard et al., 2009) [27].

Most of the patients in this study presented at an advanced stage, with stage 4 presentation being 33.1%. This is in part in accordance with the SEER reported results with 34% of patients presenting at stage 4 [8]. Similar results were described in Saudi Arabia [22]. Advanced stage (III/IV) occurring at 58.3%, is more frequent in our population than in the west, this may suggest late diagnosis related to poor socioeconomic status preventing access to healthcare.

Extranodal presentation was described in more than one-third of the patients (36.5%). Extranodal disease at diagnosis was documented in 20–30% of patients in the US (Ries et al., 2005) , and in more than 65% in patients from Saudi Arabia [22]. This disease is documented mostly in patients from France and Kuwait with 42 and 52% incidence rate respectively [22].

In this study, 18.3% (128) of patients received RT. When comparing the variables by age (≤64 vs ≥65 years), the results showed that a significantly lower percentage of patients who received RT was aged 65 years or more. In general, the indication of RT has been limited progressively to a complementary RT after chemotherapy especially in aggressive localized diseases, and this has been facilitated after the emergence of positron emission tomography (PET) scan imaging that helps selecting patients who are candidates for this approach [35]. Current evidence shows that in indolent NHL, RT may be curative in early stage disease and palliative in more advanced diseases. In aggressive NHL, RT is used to cure stage I disease after short course chemotherapy and may be given to consolidate chemotherapy response in bulky or extranodal sites. It has a valuable palliative role for aggressive lymphoma causing local symptoms in patients intolerant to chemotherapy [36]. In an attempt to review the literature where the importance of radiation therapy was assessed, several studies showed different results. In 1998, Miller et al. had investigated the superiority of three cycles of CHOP followed by involved-field radiotherapy (IFRT) to eight cycles of CHOP alone in the setting of NHL. They concluded that RT is efficacious in the setting of limited diseases and can provide curative outcomes, thus advocating its application among patients with localized lymphomas [37]. Connors et al. have also suggested that consolidative RT is an advantageous treatment and leads to decreasing the chemotherapy dose, well needed in the case of elderly patients [38]. Overall, the place of RT in the standard care of DLBCL appears debatable because some studies favors its use and others do not show definite advantage [39]. In this study, information about the bulky tumor status (defined as any mass greater than 5 cm), standardized uptake values (SUVs) on PET CT and the choice of chemotherapy before radiation in these patients are lacking. Further studies are needed to determine the characteristics of patients that had benefit from RT.

## Practical implications

NHL types and distribution presented in the study are most likely to be a reflection of the majority of patients diagnosed with NHL in Lebanon as this series encompasses all cases diagnosed in two major medical centers. This study strength include the availability of substantial and consistent medical information on a population level regarding patient’s characteristics, extracted from a clinical database that is hospital registries which provide a good coverage of NHL cases diagnosed at the time ensuring minimal selection bias. Further studies assessing the sites involved in extranodal disease should be undertaken thus investigating the impact of these sites on outcome and response to treatment of extranodal lymphomas. Moreover, studies considering socioeconomic disparities among patients are needed and that may justify for the higher percentage of people presenting at an advanced stage disease. It might be interesting to compare and assess the results among patients with lower socioeconomic class who were diagnosed mainly in the public sector.

## Limitations

After the interpretation of our results, a number of limitations needs careful discussion. First, this study is subject to the limitations of retrospective data. As this study uses the archive data records from 1984, there is concern about the accuracy of pathological diagnosis following the latest classification of NHL subtypes because it is subject to regional variations even though we tried to combine two classification systems. There is difficulty in comparing results when using different classification systems. Studies including patients diagnosed before 2001 may have codes from earlier ICD-O versions that must be converted to ICD-O-3 and have higher proportions of unclassified (e.g., lymphomas not otherwise specified) cases. In addition, the staging system that evolved during that period particularly with the introduction of 18-FDG PET CT leads us to consider the “stage migration” phenomenon. Finally, this data retrieved from two major medical centers in Lebanon accounts for the Lebanese population and results may not be generalizable to other populations, taking into account the difference in genetic background and patients characteristics. Further controlled prospective studies with larger population are necessary.

## Conclusion

This study represents a large retrospective study examining the distribution of the main NHL subtypes in Lebanon and presents a summary of the current understanding of the epidemiologic picture in a certain time frame and suggests an area of focus for future research. The results were comparable to those in other countries precisely concerning the age, gender and frequency of common lymphomas. Aggressive lymphomas types account for the majority of NHLs in Lebanon. Improvements in diagnosis with the advancement of new techniques are contributing to the better characterization of the disease. Since we lack data in Lebanon that highlight the epidemiological distribution of NHL patients, this study would supplement the existing literature. Additional studies concerning NHL in Lebanon are warranted with focus on the quality of the hematological information as well as cytogenetic and molecular features to allow internationally comparable statistics.

## Data Availability

All data generated or analyzed during this study are not publicly available to maintain the privacy of the individuals’ identities. The dataset supporting the conclusions is available upon request to the corresponding author.

## Abbreviations

NHL: Non Hodgkin Lymphoma
BCL: B-cell lymphomas
US: United States
TCL: T-cell lymphomas
OS: overall survival
IHC: Immunohistochemistry
PS: Performance status
RT: Radiotherapy
PET: Positron emission tomography
DFS: Disease free survival
IFRT: Involved-field radiotherapy
SWOG: Southwest Oncology Group
GELA: Groupe d’Etudes des Lymphomes de l’Adulte
ECOG: Eastern Cooperative Oncology Group
SPSS: Statistical Package for the Social Sciences
